# A narrative review of stigma and masking in ADHD: insights from English-language research and the Japanese cultural context

**DOI:** 10.3389/fpsyg.2026.1807337

**Published:** 2026-03-31

**Authors:** Chiharu Maeda, Laura E. Knouse, Konoka Takeda, Chisato Masudomi, Eriko Takahashi, Taisuke Katsuragawa, Hiroaki Kumano

**Affiliations:** 1 Graduate School of Human Sciences, Waseda University, Shinjuku, Japan; 2 University of Richmond, Richmond, VA, United States; 3 Faculty of Human Sciences, Waseda University, Shinjuku, Japan

**Keywords:** ADHD, stigma, masking, cultural context, Japan

## Abstract

Attention-deficit/hyperactivity disorder (ADHD) is a prevalent neurodevelopmental condition associated with impairments across educational, occupational, and social domains. Individuals with ADHD are often exposed to misunderstanding and negative evaluation, which can contribute to stigma and psychological distress. Recently, “masking”—efforts to conceal or compensate for ADHD-related characteristics to meet social expectations—has been discussed as a potential behavioral response to stigma, yet empirical research on this phenomenon remains limited. This narrative review synthesizes English-language research on public stigma and self-stigma related to ADHD and examines how stigma may be associated with masking behaviors in ADHD, drawing on conceptual insights from the literature on autism spectrum disorder (ASD) and other related fields. In addition, the review situates these processes within the Japanese cultural context, highlighting how cultural characteristics may intensify stigma and contribute to distinctive patterns of masking. By integrating cross-cultural perspectives and highlighting gaps in the current literature, this review underscores the need for ADHD-specific conceptual frameworks, culturally sensitive research, and longitudinal studies to clarify the mechanisms linking stigma and masking. These insights highlight the need for ADHD-specific and culturally sensitive frameworks to inform future research and intervention development.

## 1 Introduction

Attention deficit hyperactivity disorder (ADHD) is a neurodevelopmental disorder characterized by inattention, hyperactivity, and impulsivity (American Psychiatric Association, 2013). The disorder is associated with functional impairment in a wide variety of domains of functioning from education and employment to relationships and motor vehicle driving (Kosheleff et al., 2023). Although prevalence rates vary across studies, ADHD is reported to affect approximately 6-7% of children (Polanczyk et al., 2007) and 3–5% of adults (Polanczyk et al., 2007; Fayyad et al., 2007), suggesting a relatively high proportion of the population. In Japan, epidemiological research has also suggested that adult ADHD prevalence is around 2% (Uchiyama et al., 2012), highlighting the importance of understanding and support in educational settings, the workplace, and society at large.

Difficulties arising from ADHD have been shown to change across developmental stages (Murakami, 2017). During the elementary school years, in a context of frequent reprimands for failures stemming from the disorder’s characteristics, externalizing disorders such as intense reactions and aggressive behavior and internalizing disorders such as separation anxiety and depression are more likely to occur, increasing the risk of school maladjustment and school refusal (Murakami, 2017). During middle and high school, while self-awareness of the traits increases, behavioral problems such as avoidance of challenging situations, school refusal, and social withdrawal become more likely, in addition to externalizing and internalizing disorders (Murakami, 2017). From adolescence onward, traits like hyperactivity and impulsivity become less outwardly apparent, yet internal distress and discomfort increase (Murakami, 2017). In addition, inattentive symptoms tend to persist and are associated with impairment (Henning et al., 2024). This heightens the risk of developing internalizing disorders like anxiety and depressed mood, as well as secondary disorders such as alcohol dependence, substance abuse, and social withdrawal (Murakami, 2017).

Individuals with ADHD often struggle to engage in socially desirable behaviors due to difficulties with attention and behavioral regulation (Parke et al., 2021). Consequently, they may face misunderstanding and negative evaluations from others (Beaton et al., 2022). Such misunderstandings and negative assessments may contribute to the formation of stigma surrounding ADHD, both from others and within the individual, becoming factors that may heighten psychological distress and the risk of secondary disorders for those affected.

### 1.1 Stigma and ADHD

Stigma encompasses fixed ideas about and prejudices held toward individuals or groups possessing specific attributes, as well as discriminatory actions based on these prejudices (Corrigan and Watson, 2002). There are multiple types of stigma, including public stigma, prejudice and discriminatory bias held by society at large toward specific attributes; self-stigma, which is the internalization of public stigma by the individual themselves, leading to negative self-evaluation; associative stigma, which is prejudice or discriminatory attitudes directed at people associated with the stigmatized individual, such as family or close associates; and structural stigma, which occurs when institutional policies and cultural norms systematically disadvantage people with the stigmatized attribute.

Stigma has been noted to exert diverse effects in ADHD, broadly impacting psychosocial functioning through delayed treatment and support, restricted social participation, and diminished self-esteem and self-efficacy (Mueller et al., 2012). In recent years, attention has increasingly focused on “masking” behaviors in ADHD as a potential behavioral response to stigma. Although stigma-specific programs targeting ADHD have yet to be developed, it has been suggested that stigma-reduction strategies incorporating the three elements of protest, education, and contact proposed by Corrigan and Shapiro (2010) should be integrated into comprehensive psychoeducational intervention plans (Bussing and Mehta, 2013).

### 1.2 Stigma and ADHD masking behavior

Recent research indicates that stigma affects not only psychosocial functioning but also a stigmatized persons’ levels of openness and self-expression. Specifically, for people with autism spectrum disorder (ASD), “social camouflaging”—the act of concealing ASD traits—increases with higher levels of stigma toward ASD (Perry et al., 2022) and has been reported to heighten risk for anxiety and depression (Hull et al., 2021). Regarding ADHD, “ADHD masking” refers to the compensatory strategies and efforts individuals engage in—based on their own cognitions and beliefs—to conceal or compensate for ADHD-related characteristics in order to meet demands in social environments, such as tasks or communication (Maeda et al., 2024a). Such behaviors may lead to increased psychological burden and avoidance of interpersonal interactions (Maeda et al., 2024a). Maeda et al. (2024a) further suggest that ADHD masking behaviors may be driven by external factors such as negative attitudes and prejudices from others, as well as internal factors such as individuals’ negative beliefs about themselves. In other words, both public stigma and self-stigma may influence the development and maintenance of masking. Most research on stigma-related masking behaviors has focused on ASD, and only a few have examined ADHD. Moreover, although ADHD masking has become a widely discussed topic within online and peer-based communities of adults who identify as having ADHD and the popular press (Stavraki, 2024), academic research has not yet adequately engaged with these lived experiences. This gap further highlights the need for systematic empirical attention to masking in ADHD. To this end, it may be useful to consider how the camouflaging literature in ASD might inform theorizing about masking in ADHD. Although masking in ADHD may differ qualitatively from camouflaging in ASD, the ASD literature provides a useful heuristic framework for generating hypotheses about stigma-related masking processes in ADHD.

### 1.3 Cultural context of ADHD stigma and masking: challenges in Japan

The form and intensity of stigma are influenced by cultural context (Krendl and Pescosolido, 2020). Specifically, in cultures with strong collectivist values, negative evaluations are more likely to arise toward deviations from social norms (Furnham and Chan, 2004). While Japan has seen a recent strengthening of individualistic aspects, it still possesses collectivist cultural characteristics that emphasize conformity to the group and adherence to discipline (Ogihara, 2017). Therefore, examination grounded in Japan’s unique cultural context is required, yet research on ADHD stigma in Japan remains scarce. More broadly, empirical research considering cultural background is needed on the relationship between stigma and masking behaviors.

### 1.4 Purpose of this review

This review first examines ADHD-related public stigma and self-stigma, which may influence ADHD masking. Next, drawing on findings from the ASD literature, we describe potential links between ADHD stigma and masking. Finally, we discuss cultural characteristics of stigma surrounding ADHD in Japan and implications for masking, while identifying directions for future research. Ultimately, the aim is to present a foundational perspective for understanding and supporting those affected by ADHD stigma in Japan and to encourage additional research on ADHD stigma and masking across cultures.

### 1.5 Literature identification

Relevant literature was identified through searches of English-language and Japanese-language academic databases. For English-language literature, searches were conducted using PubMed, which is widely used in biomedical and psychological research, whereas Japanese-language literature was searched using J-STAGE and CiNii Articles, which are widely used academic databases in Japan. Broad search terms related to stigma and masking were used to identify potentially relevant studies, and no specific restrictions were placed on publication years to capture a wide range of relevant literature. In addition, the reference lists of the identified articles were examined, and further relevant studies were identified through manual searches. The titles, abstracts, and full texts of the identified articles were independently reviewed by the first author and two graduate students specializing in clinical psychology to determine whether they included descriptions related to stigma or masking behaviors relevant to the aims of this narrative review.

## 2 Stigma and masking of ADHD in English-language countries

In this narrative review, English-language studies were examined to complement the discussion of stigma and masking in ADHD. In particular, the analysis drew on the broader literature on social camouflaging in ASD, which has been more extensively developed, to provide conceptual context for understanding masking-related behaviors.

### 2.1 Public stigma

Public stigma toward ADHD refers to prejudice and discrimination toward people with ADHD within society. Research on public stigma toward ADHD has developed within the broader context of stigma research on mental disorders. Its theoretical foundation relies on sociological theories, such as the concept of stigma proposed by Goffman (1963) and the model of stigma formation processes by Link and Phelan (2001). Consequently, frameworks focusing on social processes like labeling, stereotyping, and social segregation are frequently used to examine the factors contributing to public stigma formation.

Misconceptions that ADHD results from laziness or lack of effort remain deeply rooted in society (Mueller et al., 2012). In addition, media portrayals and online discourse have often emphasized themes of overdiagnosis and medication misuse, further reinforcing deficit-based views of ADHD (Bisset et al., 2022). Although research directly investigating public stigma toward ADHD remains limited, existing findings indicate that such stigma can manifest as skepticism about the legitimacy of ADHD and blame directed at individuals with ADHD and their families (Visser et al., 2024).

Public stigma toward ADHD—including skepticism about the disorder, blame directed at individuals with ADHD and their families—can give rise to a lack of appropriate support and discriminatory treatment (Visser et al., 2024). Moreover, this stigma may manifest in broader and more diverse harms, such as rejection, conflict, and social exclusion within educational settings (Visser et al., 2024). Such adverse experiences can negatively affect identity development and psychological well-being among individuals with ADHD (Visser et al., 2024). In line with these qualitative findings, quantitative evidence from Metzger and Hamilton (2020) shows that students labeled with ADHD are consistently rated by their teachers as performing below grade level in academic subjects—including math and reading—even when their objective test scores are controlled for. This suggests that teachers’ biased perceptions—possibly rooted in stereotypes about ADHD—may lead to systematic underestimation of students’ academic potential and unfair treatment in the classroom, beyond their actual cognitive or behavioral characteristics. Extending beyond educational settings, individuals with ADHD also face substantial stigma-related challenges in the workplace. Many report that ADHD symptoms hinder their job performance, prevent them from realizing their potential, and leave them feeling unable to meet their own standards (Fuermaier et al., 2021). A review by Küpper et al. (2012) further indicates that adults with ADHD experience markedly higher unemployment rates and reduced work performance compared to those without ADHD, highlighting significant consequences for occupational health and functioning. Even when individuals disclose their diagnosis to seek support, their supervisors or managers may lack knowledge about ADHD or fail to understand the specific difficulties faced by adults with the condition (Oscarsson et al., 2022). As a result, workers with ADHD often experience dilemmas about disclosure and harbor concerns about stigma, discrimination, and negative evaluation in the workplace (Oscarsson et al., 2022). Taken together, these findings demonstrate that public stigma toward ADHD operates across multiple domains of daily life, shaping educational and occupational outcomes and imposing significant psychological and social burdens on affected individuals.

### 2.2 Self-stigma

Research on ADHD self-stigma has developed within the broader context of stigma research on mental illness, similar to public stigma studies. Its foundation lies in the theory proposed by Corrigan and Watson (2002), which posits that self-stigma arises when individuals with ADHD internalize public stigma (Visser et al., 2024).

Individuals with ADHD are more likely to experience difficulties and failures in school and home environments, resulting in frequent exposure to reprimands and negative feedback (Beaton et al., 2022). Such personal experiences may contribute to negative self-perceptions and the deepening of self-stigma. Indeed, self-stigma during childhood and adolescence is characterized by negative self-evaluation and self-blame associated with perceived differences from peers (McKeague et al., 2015), which may lead to reduced self-esteem, psychological distress, and diminished motivation and ability to pursue goals (Jelinkova et al., 2024). Indeed, empirical research has shown that self-stigma among individuals with ADHD is associated with lower self-esteem, increased depression and emotional difficulties, and poorer social adjustment. For example, a psychometric study using the ADHD Stigma Questionnaire (ASQ) found that higher stigma scores among individuals with ADHD correlated with worse emotional symptoms, lower self-esteem, and clinical maladjustment (Kellison et al., 2010). More recently, a survey of young people with ADHD and their parents found that increased self-stigma was significantly associated with lower self-esteem and poorer mental health (Jelinkova et al., 2024). Together, these studies collectively support the conclusion that self-stigma may contribute to depression, maladjustment, and loneliness in ADHD. Furthermore, it has been noted that self-stigma significantly impacts the process of receiving treatment and support for ADHD, including reduced help-seeking and treatment adherence behaviors, limited access to treatment and support, and resulting delays in diagnosis (Visser et al., 2024; Mueller et al., 2012).

### 2.3 ADHD masking: empirical evidence and conceptual challenges

To date, only a few empirical studies have investigated masking in adult ADHD as a primary research topic. Wurth et al. (2025) found that, on average, adults with ADHD agreed with the statement, “I feel I have to mask my personality when I am at school, work, and at home.” van der Putten et al. (2024) examined camouflaging behaviors in adults with ADHD using the CAT-Q (Hull et al., 2019), a measure originally developed to assess camouflaging in individuals with autism. The study compared 105 adults with ADHD with matched groups of 105 adults with autism and 105 neurotypical adults (van der Putten et al., 2024). Results showed that adults with ADHD reported significantly higher overall camouflaging than neurotypical participants, particularly in behaviors aimed at fitting in with others in social situations (van der Putten et al., 2024). However, they did not differ from neurotypical adults in strategies focused on compensating for social and communication difficulties or in behaviors intended to hide ASD traits (van der Putten et al., 2024). In contrast, compared with adults with autism, individuals with ADHD showed significantly lower overall camouflaging and less frequent use of compensatory strategies and fitting-in behaviors (van der Putten et al., 2024). Regression analyses further indicated that ASD traits, but not ADHD traits, significantly predicted camouflaging scores (van der Putten et al., 2024). Based on these findings, the authors concluded that adults with ADHD engage in more camouflaging than neurotypical adults but less than adults with autism (van der Putten et al., 2024).

Importantly, these findings must be interpreted considering a key methodological limitation. The authors emphasized that the CAT-Q was specifically designed to capture camouflaging behaviors characteristic of ASD. As such, it may not adequately reflect the situations, symptoms, or strategies relevant to masking in adults with ADHD. This measurement issue highlights a broader challenge in the field: the lack of ADHD-specific conceptualizations and assessment tools for masking. Taken together, further tailored research is necessary for the ADHD population.

Knowledge regarding masking behaviors in ADHD remains limited. A narrative review by Maeda et al. (2024a) found no studies that explicitly positioned “ADHD masking” as a primary construct. References to masking behaviors were typically confined to limited sections of the text and were commonly embedded within studies addressing related but distinct topics. Consequently, descriptions functionally corresponding to masking had to be extracted at the sentence level from studies focusing on these related topics (Maeda et al., 2024a). The findings of this review suggest that ADHD masking may be associated with short-term benefits, such as reducing ADHD-related difficulties and facilitating social adaptation, while also being linked to potential long-term disadvantages, including increased psychological burden and avoidance of interpersonal interactions (Maeda et al., 2024a).

### 2.4 How might ADHD stigma influence masking behavior and its consequences?

Given these empirical and conceptual gaps, insights from related fields may help inform theorizing about ADHD masking. With respect to the potential link between stigma and masking in ADHD, while empirical research remains limited, several studies have examined the relationship between stigma and camouflage behaviors in ASD. Perry et al. (2022) investigated this link based on social identity theory. Their findings indicated that higher perceived stigma toward ASD was associated with higher levels of camouflage behavior (Perry et al., 2022). Zhuang et al. (2024) proposed an integrated model of camouflage motivation, examining the relationship between proximate and distal sociocultural factors—including stigma—alongside individual psychological factors. The results suggested that stigma may indirectly influence camouflaging behavior through individual psychological factors such as fear of negative evaluation and self-esteem among individuals with ASD (Zhuang et al., 2024).

In ADHD, although evidence is currently based largely on theoretical proposals rather than direct empirical tests, stigma has similarly been suggested to contribute not only to reduced self-esteem and loneliness, but also to masking behaviors (Visser et al., 2024). Based on findings from camouflaging research in ASD, it can be inferred that higher perceived stigma may also be associated with increased masking behaviors in ADHD. Furthermore, considering that individuals with ADHD are prone to experiencing failure and negative evaluations in many settings like school and home, it is plausible that masking behaviors in ADHD, similar to ASD, may form and be maintained through psychological factors such as fear of negative evaluation.

Taken together with prior findings on the consequences of masking, these considerations suggest that stigma and masking are likely to interact in complex ways, potentially amplifying psychological burden and influencing mental health and social functioning in ADHD. However, the mechanisms underlying these processes remain poorly understood. Accordingly, future research is needed to clarify how stigma contributes to masking in ADHD and how this interplay affects mental health and social adjustment across different contexts.

## 3 Stigma and masking regarding ADHD in Japan

Although research on ADHD stigma and its association with masking behavior is currently limited, prior studies have demonstrated substantial cross-cultural variation in mental health stigma (Krendl and Pescosolido, 2020), providing strong reasons to suspect that ADHD masking behavior is influenced by cultural context. While the concept of ADHD masking remains underdeveloped, studies conducted in Japan provide context-specific insights into how such behaviors may be experienced and interpreted within a particular sociocultural setting.

In this section, we focus primarily on Japanese-language studies examining stigma and masking in ADHD, particularly qualitative interview research involving individuals with ADHD. To complement this discussion, relevant literature on social camouflaging in ASD, as well as research on stigma and coping in other mental health conditions, is also considered.

Based on these perspectives, this section discusses cultural factors that may influence stigma and masking behaviors among individuals with ADHD in the Japanese context.

### 3.1 ADHD public stigma and self-stigma in Japan

Stigma surrounding ADHD in Japan is not merely a negative evaluation of individual traits but is deeply intertwined with sociocultural values and normative expectations. Contemporary Japanese society is characterized by the coexistence of increasing individualistic values and the persistence of collectivistic norms (Ogihara, 2017). Although individualism has risen over time, collectivistic orientations emphasizing social harmony and conformity remain influential (Ogihara, 2017), creating potential tensions in behavioral expectations and interpersonal relationships. In Japanese society, where actions like “reading the air” and “not causing trouble for others” are valued, behaviors stemming from ADHD traits such as inattention or impulsivity may be interpreted as “sloppiness,” potentially leading to unfair criticism (Kiuchi, 2016). Thus, the stigma surrounding ADHD in Japan may be reinforced and maintained within the context of moral judgments and social expectations to a greater extent than in other cultural contexts.

Such cultural values also significantly influence the formation and maintenance of stigma. In Japan, there is a strong tendency to attribute mental illnesses such as depression and schizophrenia to “weak character,” which has been pointed out to lead to delayed requests for assistance, treatment discontinuation, and poor medication adherence (Yokoya et al., 2018; Oguchi, 2022). Similarly, for ADHD, symptoms may be attributed to personal character flaws or lack of effort, potentially contributing to discrimination toward people with ADHD and hindering appropriate diagnosis and support. Furthermore, Someki et al. (2018) reported that Japanese university students, compared to their American counterparts, were more likely to attribute ASD causes to family environments such as “child-rearing” and exhibited higher rates of negative attitudes toward marrying individuals with ASD. Against this cultural backdrop, stigma surrounding ADHD-related behaviors persists strongly in Japan and may promote the internalization of stigma by individuals with the disorder. Consequently, there is concern that both public and internalized stigma could lead to multifaceted adverse effects on psychosocial functioning, such as inhibiting requests for assistance and lowering self-esteem.

### 3.2 Potential impact of stigma on ADHD masking in Japan

To date, no empirical studies have directly examined the relationship between ADHD stigma and masking in Japan. However, regarding ASD in Japan, it has been reported that higher perceived stigma correlates with increased social camouflage behaviors (Tamura et al., 2025). This trend is consistently demonstrated in English-language studies as well (Perry et al., 2022).

Notably, in Japan, excessive social camouflage is associated with poorer mental health, but so is insufficient camouflage (Oshima et al., 2024). Multiple factors are thought to underlie the negative consequences of both excessive and insufficient camouflaging, including low social acceptance, insufficient understanding of developmental characteristics, and Japan’s unique cultural values emphasizing conformity and uniformity (Oshima et al., 2024). While excessive camouflage behaviors may cause psychological burden and fatigue, insufficient camouflaging can also lead to social exclusion and isolation in Japan’s conformity-oriented society. Thus, individuals may face a dual risk structure: in which both doing too much and too little camouflaging can result in negative outcomes.

A qualitative interview study with adults diagnosed with ADHD in Japan has shown that masking behaviors are shaped by multiple sociocultural and psychological factors, such as criticism or reprimands from teachers and peers as well as self-reflection based on repeated failures or social difficulties (Maeda et al., 2024b). Within this process, both public stigma and self-stigma are likely to play fundamental roles. Negative social evaluations and normative pressures within the Japanese sociocultural context may promote efforts to hide visible signs of ADHD, while internalized negative beliefs may further reinforce the need to suppress one’s characteristics to avoid rejection.

Within this Japanese interview study, masking behaviors serve both adaptive and maladaptive functions (Maeda et al., 2024b). On the adaptive side, masking may help individuals navigate daily life, meet social expectations, and maintain smoother interpersonal interactions (Maeda et al., 2024b). However, when masking becomes excessive, individuals experience constant behavioral monitoring, emotional exhaustion, reduced self-efficacy, and a decline in overall well-being (Maeda et al., 2024b). Conversely, drawing from research on social camouflaging, insufficient masking may also present significant risks in sociocultural contexts such as those found in Japan, where expectations for social conformity are often emphasized. Taken together, these dynamics suggest that individuals with ADHD in Japan face a dual-risk structure, in which both excessive and insufficient masking can lead to adverse outcomes. In ADHD, inattentive behaviors are particularly vulnerable to moral interpretation as laziness or lack of effort in the Japanese context, where diligence and conformity are strongly emphasized. As a result, ADHD-related difficulties may be viewed as moral or personal failures rather than neurodevelopmental differences, reinforcing both public and self-stigma. This ADHD-specific form of stigma is likely to play a critical role in shaping masking behaviors in Japan, distinguishing ADHD from other neurodevelopmental conditions such as ASD. This indicates a broader structural problem, in which stigma and cultural values emphasizing conformity mutually reinforce masking behaviors and ultimately exacerbate psychological burden.

Given the above, a key challenge for Japanese society moving forward is how to build and expand environments that can accommodate individuals with diverse behavioral patterns and characteristics. Simultaneously, for individuals with ADHD themselves, it is important to adopt a perspective of flexible adjustment tailored to their own needs, based on psychological costs or benefits and situational context, rather than a binary choice of whether or not to engage in masking behaviors. Specifically, adjustments rooted in individual life contexts are required. This includes selecting situations where masking is necessary versus unnecessary based on personal psychological burden and surrounding circumstances, or implementing masking with content and frequency suited to the individual. Thus, a perspective that balances expanding social acceptance with adaptive adjustments by the individual is considered essential for maintaining and improving the mental health of individuals with ADHD.

## 4 Limitations of this review and the current state of the literature

This narrative review was designed to provide an integrative overview of stigma related to ADHD and its potential association with masking behaviors and cultural context, rather than to produce a comprehensive systematic synthesis. As such, we did not conduct formal assessments of methodological quality or risk of bias in the included studies. In addition, although efforts were made to identify relevant literature, this review does not claim to be exhaustive, and it is possible that some empirical studies or theoretical papers were not captured.

A further limitation concerns the nature of the existing literature on ADHD stigma and masking. Research on ADHD stigma remains fragmented across disciplines, leading to variability in conceptual definitions and measurement approaches. Many studies rely on general mental-illness stigma frameworks, which may not fully capture ADHD-specific mechanisms such as misunderstanding of the disorder, perceptions of controllability, or stereotypes about effort and responsibility.

Similarly, empirical research on ADHD masking is at an early stage. There is no unified operational definition or validated measurement tool, and references to masking often appear only incidentally within broader studies, limiting the strength of inferences that can be drawn. Although research on camouflaging in ASD has developed more robust conceptual frameworks and measurement instruments, the application of these ASD-based models to ADHD remains largely inferential, as the specific mechanisms and manifestations of masking may differ between the two conditions.

## 5 Future research

Future research should first aim to strengthen the conceptual and methodological foundations of ADHD stigma and masking research. For stigma, this includes developing ADHD-specific theoretical models that differentiate public stigma, self-stigma, associative stigma, and structural stigma, and clarifying how these forms interact with cultural norms and institutional practices. For masking, establishing a precise conceptual definition and developing reliable measurement tools are urgently needed to enable empirical investigation.

Longitudinal and multi-method studies are essential to examine the mechanisms linking stigma and masking over time. Such designs would help clarify the relationships between stigma and masking, their psychological and functional impacts, and how these processes unfold across developmental stages from childhood through adulthood.

Cultural and cross-cultural studies represent another important direction. Research conducted in Japan and other non-Western societies will help clarify how cultural values such as conformity, collectivism, and role expectations influence both stigmatizing attitudes and masking behaviors. Research within nations, including the United States, should also investigate how ADHD stigma and masking function within subcultures and how factors such as the gender of the person with ADHD interact with culture to impact perceptions, stigma, and masking.

In addition, future research should incorporate contextual factors—including school policies, workplace practices, healthcare accessibility, and family environments—to understand how structural stigma shapes individual-level masking strategies and psychological outcomes.

Finally, intervention studies are needed to evaluate strategies that reduce stigma (e.g., education, contact-based programs, policy-level interventions) and to examine whether reducing stigma can help decrease maladaptive masking and improve mental health and social adjustment among individuals with ADHD.

## 6 Conclusion

This review illustrates that stigma toward ADHD—including public and self-stigma—plays a substantial role in shaping individuals’ psychological well-being, social participation, and access to support. Although empirical research on ADHD masking remains limited, existing evidence suggests that stigma may contribute to the formation and maintenance of masking behaviors more broadly. However, this dynamic may be particularly pronounced in cultural settings such as Japan, where conformity and adherence to social norms are strongly emphasized. In such environments, individuals with ADHD may face a dual-risk structure in which both excessive and insufficient masking can negatively affect mental health and social functioning.

By integrating findings from prior research and situating them within broader sociocultural contexts, this review underscores the urgent need for empirical studies that clarify the pathways linking stigma and masking and identify effective forms of support. Building such an evidence base will be essential for informing interventions, public policy, and clinical practice. Ultimately, advancing these efforts will facilitate the development of social and clinical environments that minimize stigma-related pressures, help prevent maladaptive forms of masking, and enable individuals with ADHD to participate in daily life with greater psychological safety and inclusion.
